# Spesolimab for generalized pustular psoriasis: a review of two key clinical trials supporting initial US regulatory approval

**DOI:** 10.3389/fimmu.2024.1359481

**Published:** 2024-07-22

**Authors:** Eran C. Gwillim, Anna J. Nichols

**Affiliations:** ^1^ Dr. Phillip Frost Department of Dermatology and Cutaneous Surgery, University of Miami Miller School of Medicine, Miami, FL, United States; ^2^ Jackson Health System, Miami, FL, United States

**Keywords:** generalized pustular psoriasis, spesolimab, clinical trial, efficacy, safety

## Abstract

Generalized pustular psoriasis (GPP) is a chronic, rare, and potentially life-threatening inflammatory disease, characterized by the rapid and widespread eruption of small, sterile pustules with surrounding skin erythema. Abnormal signaling of the interleukin-36 (IL-36) pathway appears to have a central role in GPP immunopathology, and provides a rational therapeutic target. Spesolimab is a first-in-class humanized monoclonal antibody that binds specifically to the IL-36 receptor, and antagonizes IL-36 signaling. Spesolimab obtained regulatory approval in the United States (US) in September 2022 for use in the treatment of GPP flares in adults, and was subsequently approved for GPP flare treatment in many other countries across the world. Recently, regulatory approval was granted for subcutaneous dosing of spesolimab for treatment of GPP when not experiencing a flare. Here, we review data from two key clinical trials that supported the initial US regulatory approval; namely, the phase 1 proof-of-concept trial (ClinicalTrials.gov ID, NCT02978690), and Effisayil^™^ 1 (NCT03782792), which remains the largest and only randomized clinical trial in patients experiencing GPP flares published to date. In the phase 1 proof-of-concept trial, a Generalized Pustular Psoriasis Physician Global Assessment (GPPGA) score of 0 or 1 (clear or almost clear skin) was attained in 5/7 (71%) patients by week 1 and in all 7 patients by week 4; and the mean percent improvement in the Generalized Pustular Psoriasis Area and Severity Index (GPPASI) score from baseline was 59.0% at week 1, 73.2% at week 2, and 79.8% at week 4. In Effisayil^™^ 1, a GPPGA pustulation subscore of 0 (no visible pustules) was achieved in 19/35 (54%) patients receiving spesolimab at the end of week 1, versus 1/18 (6%) receiving placebo (difference, 49 percentage points; 95% confidence interval [CI], 21 to 67; P<0.001); and a GPPGA total score of 0 or 1 was achieved by 15/35 (43%) patients in the spesolimab group, versus 2/18 (11%) patients in the placebo group (difference, 32 percentage points; 95% CI, 2 to 53; P = 0.02). Infections at week 1 were reported in 6/35 (17%) patients receiving spesolimab and in 1/18 (6%) patients receiving placebo. These data demonstrate the efficacy and safety of spesolimab in providing rapid and sustained clinical improvement for patients with GPP flares, which translates into improved quality of life, by offering a targeted therapy for GPP.

## 1 Introduction

Generalized pustular psoriasis (GPP) is a chronic, rare, potentially life-threatening, multisystem inflammatory condition that is frequently painful and distressing (1) and has a detrimental effect on the quality of life of affected individuals (2–5). Until recently, no GPP-specific treatments for disease flares were approved in the United States (US) or Europe, and available systemic treatment options for patients with GPP consisted primarily of agents used to treat plaque psoriasis (i.e., off-label use) (6). In Japan, several biological therapies are approved for use in GPP, including monoclonal antibodies against tumor necrosis factor (TNF)-α (adalimumab, infliximab, and certolizumab pegol), interleukin (IL)-17 (IL-17A: secukinumab and ixekizumab; IL-17 receptor: brodalumab), and IL-23 (risankizumab and guselkumab) [reviewed in (6, 7)]. However, the supporting data are somewhat limited and mainly arise from case reports and/or small open-label clinical trials (7). Consequently, there remains an unmet need for agents to control GPP flares and provide long-term maintenance therapy to prevent further relapse. Ideally, such therapies would demonstrate rapid time to clear GPP flares and reduce systemic manifestations and disease recurrence (1). However, the low prevalence of GPP and the relapsing–remitting course of the disease have made it difficult to obtain good-quality evidence on the efficacy and safety of treatment candidates. These issues, plus the potential severity of an acute flare, present significant challenges in undertaking randomized placebo-controlled clinical trials in the GPP population (8).

GPP usually occurs in adults, although children and infants may be affected (1). The rarity of GPP makes its diagnosis challenging. Most healthcare providers are unlikely to encounter many affected patients (9), and may confuse the associated systemic symptoms with an infectious disorder. Estimates of GPP prevalence show considerable variation, ranging from approximately 2 to 120 cases per million persons (10–13). Emerging clinical, histological, and genetic data have revealed that GPP is a distinct entity from plaque psoriasis and that it warrants a separate diagnosis (14–18), although these two conditions may occur concurrently (14). GPP is characterized by the rapid and widespread eruption of small, sterile pustules with surrounding skin erythema (19), and the pustules may coalesce into larger lesions (termed “lakes of pus”). Common systemic symptoms include fever, skin pain, and malaise (20). Extracutaneous manifestations may also occur, including arthritis and cholangitis, which adds to the disease burden (2, 21). The course of GPP is highly variable. It may be relapsing–remitting, with relapses of an idiopathic nature or following exposure to certain triggers (such as infection, stress, pregnancy, and in association with certain drugs, e.g. withdrawal of systemic corticosteroids), or it may be more persistent, with symptoms lasting for several months (14, 20). Mortality data in patients with GPP are limited, but a review of recent studies reported that deaths attributable to GPP flares occurred in 5–10% of patients with GPP, commonly due to sepsis or septic shock (3).

The precise cause of GPP remains unknown, although abnormal IL-36 signaling appears to have a central role in its immunopathology and provides a rational therapeutic target (15, 17, 22–27). Loss-of-function mutations in the *IL36RN* gene—which encodes the IL-36 receptor antagonist (IL-36Ra), a negative regulator of the IL-36 pathway—have been reported in sporadic and familial cases of GPP (8, 17, 22). *IL36RN* mutation frequency is variable [up to 82% in familial groups (23) and 20–75% in sporadic case series (17, 24, 28, 29)]. A systematic review and meta-analysis of 683 patients with GPP reported that *IL36RN* mutation was strongly related to GPP without plaque psoriasis leading to early-onset GPP (30). Furthermore, a single-nucleotide polymorphism (c.115 + 6T>C) of the *IL36RN* gene had a significant role in GPP vulnerability (30). Mutations in other genes associated with GPP, also linked to the IL-1/IL-36 pathway, have been identified, including caspase-activating recruitment domain member 14 (*CARD14*), adaptor protein complex 1 subunit sigma 3 (*AP1S3*), TNFAIP3-interacting protein 1 (*TNIP1*), and serpin family A member 3 (*SERPINA 3*) (31).

Spesolimab (SPEVIGO^®^, Boehringer Ingelheim Pharmaceuticals, Inc., Ridgefield, CT, USA) is a first-in-class humanized monoclonal antibody that binds specifically to the IL-36 receptor to antagonize IL-36 signaling, and inhibit downstream activation of proinflammatory and profibrotic pathways (32, 33). Spesolimab was approved by the US Food and Drug Administration (FDA) in September 2022 to treat GPP flares in adults (34), and was subsequently approved for GPP flare treatment in other regions across the world, including Japan, China, and the European Union (32, 35). Spesolimab to treat GPP flares is administered as a single 900-mg dose via intravenous (IV) infusion over 90 minutes, with the option of a second 900-mg dose IV given 1 week later if symptoms persist. Recently, regulatory approval was granted for subcutaneous dosing of spesolimab for treatment of GPP when not experiencing a flare (details can be found in the label) (33).

The aim of this article is to review the two key clinical trials that supported the initial US regulatory approval and contextualize the potential impact of spesolimab in providing targeted therapy for patients with GPP flares.

## 2 Key clinical trials with spesolimab

**Figure 1:**
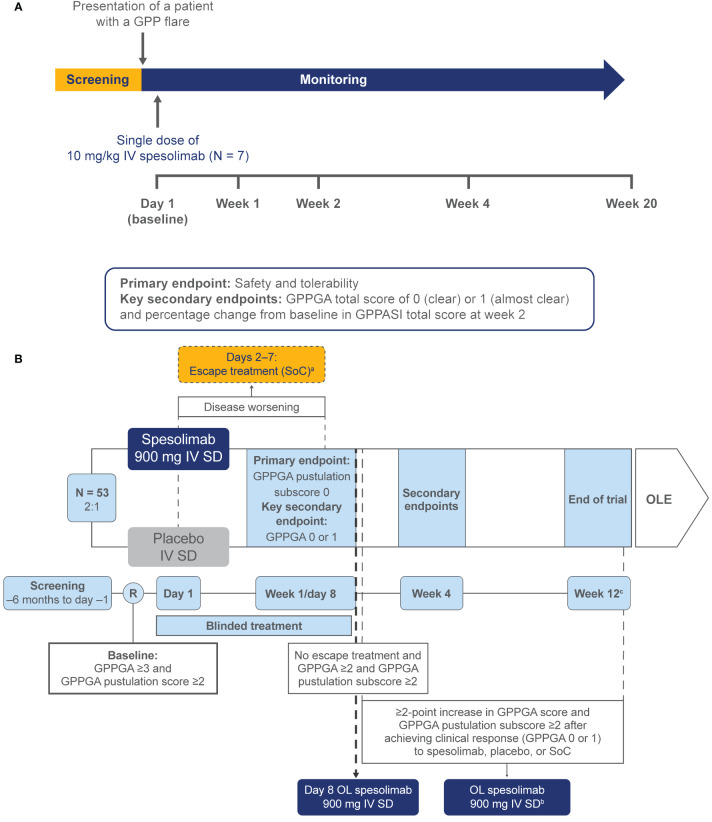
Spesolimab clinical trial designs. **(A)** Phase 1 proof-of-concept trial (36). GPPGA, Generalized Pustular Psoriasis Physician Global Assessment; GPPASI, Generalized Pustular Psoriasis Area and Severity Index; IV, intravenous. **(B)** Phase 2 Effisayil^™^ 1 trial (38). ^(a)^ Days 2–7: Escape treatment (SoC) may be offered in case of disease worsening defined as worsening of clinical status or GPP skin and/or systemic symptoms as defined by the investigator. ^(b)^ After day 8 to week 12: Only one rescue dose with OL spesolimab is permitted if a patient who has previously achieved GPPGA 0/1 to initial treatment, either with spesolimab or placebo at day 1 or escape medication or OL spesolimab at day 8, experiences a recurrence of a GPP flare (≥2-point increase in the GPPGA score and the pustular component of GPPGA ≥2). Subsequent flares will be treated with SoC per the physician’s choice. ^(c)^ Patients who do not require rescue treatment with OL spesolimab after day 8 are to be followed until week 12 (EoT) prior to entering into the OLE trial. Patients who receive rescue treatment with OL spesolimab between weeks 2 and 6 are to be followed until week 12 (EoT) prior to entering the OLE trial. If at week 12 they qualify to enter the OLE trial, the EoT will be considered for these patients. Patients who do not qualify to enter the OLE trial are to be followed for 16 weeks (EoT/weeks 16–28) after the last dose of trial medication, which is the latest time point of trial medication given during the trial (e.g., day 1, day 8 if OL spesolimab is given, rescue with OL spesolimab if given). EoT, end of trial; GPP, generalized pustular psoriasis; GPPGA, Generalized Pustular Psoriasis Physician Global Assessment; IV, intravenous; OL, open-label; OLE, open-label extension; R, randomization; SD, single dose; SoC, standard of care. Reprinted with permission from Bachelez H, Choon SE, Marrakchi S, Burden AD, Tsai TF, Morita A, et al. Efficacy and safety of BI 655130, an anti-interleukin-36 receptor antibody, in patients with acute generalized pustular psoriasis. 27^th^ European Academy of Dermatology and Venereology (EADV) Congress; September 12–16, 2018, Paris, France. Abstract 4492 and Oral presentation. Copyright 2018 with permission from Dr H Bachelez. From *N Engl J Med*. Bachelez H, Choon SE, Marrakchi S, Burden AD, Tsai TF, Morita A, et al. Trial of spesolimab for generalized pustular psoriasis. 2021;385:2431–40. Copyright ^©^ (2021) Massachusetts Medical Society. Reprinted with permission from Massachusetts Medical Society.

Published clinical trial experience with spesolimab primarily consists of a phase 1 proof-of-concept trial (36) and a phase 2 randomized, placebo-controlled trial (Effisayil^™^ 1) (37, 38) [**Figures 1A, B** (36, 38)]. Spesolimab efficacy was assessed via the Generalized Pustular Psoriasis Physician Global Assessment (GPPGA) and the Generalized Pustular Psoriasis Area and Severity Index (GPPASI) (39) [**Table 1** (36, 38)]. These tools were created with the help of leading global experts in GPP and psoriasis vulgaris (37). The GPPGA is a physician-based assessment of the severity of pustules, erythema, and scaling of GPP lesions and is a modified version of the Physician (or Investigator) Global Assessment. The GPPGA has been validated clinically (40), and minimal clinically important differences (MCIDs) have been defined (41). The GPPASI is an adaptation of the Psoriasis Area and Severity Index (42), in which the induration component is replaced by a pustule component. Clinical validation of the GPPASI was published recently (40). Biomarker data were also collected. Patient-reported outcome (PRO) instruments were utilized, and their correlation with efficacy was assessed. PRO questionnaires included the Dermatology Life Quality Index (DLQI), Functional Assessment of Chronic Illness Therapy – Fatigue (FACIT-F) scale, which assesses the effect of fatigue on daily activities; the Psoriasis Symptom Scale (PSS), which assesses pain, redness, itching, and burning symptoms during the past 24 hours; and the pain visual analog scale (VAS).

**Table 1:** GPPGA^a^ and GPPASI^b^ (36, 38).

Score	Pustules	Erythema	Scaling
0	No visible pustules	Normal or postinflammatory hyperpigmentation	No scaling or crusting
1	Low-density occasional small discrete pustules (non-coalescent)	Faint, diffuse pink, or slight red	Superficial focal scaling or crusting restricted to periphery of lesions
2	Moderate-density grouped discrete small pustules (non-coalescent)	Light red	Predominantly fine scaling or crusting
3	High-density pustules with some coalescence	Bright red	Moderate scaling or crusting covering most or all lesions
4	Very-high-density pustules with pustular lakes	Deep fiery red	Severe scaling or crusting covering most of all lesions

GPP, generalized pustular psoriasis; GPPASI, Generalized Pustular Psoriasis Area and Severity Index; GPPGA, Generalized Pustular Psoriasis Physician Global Assessment.

a GPPGA: Erythema, pustules, and scaling of all psoriatic lesions are scored from 0 to 4. Each component is scored on a 5-point scale (ranging from 0 [least severe] to 4 [most severe]), the average for each is calculated, and the final GPPGA score is determined. A lower score indicates a lesser severity, with 0 relating to “clear” and 1 relating to “almost clear”. To receive a score of 0 or 1, the patient should be afebrile in addition to the skin presentation requirements.

b GPPASI: This tool provides a numeric scoring for the patient’s overall GPP disease state, ranging from 0 to 72; it is a linear combination of the percent of skin surface area affected (body region area score) and the severity. It is scored on a 5-point scale (ranging from 0 [least severe] to 4 [most severe]) of erythema, pustules, and scaling over 4 body regions (head, upper limb, trunk, and lower limb). Individual score per body region = body region factor (head = 0.1, upper limb = 0.2, trunk = 0.3, lower limb = 0.4) × body region area score × sum of component severity scores in body region. Total GPPASI score = sum of individual score from all body regions.

From *N Engl J Med*. Bachelez H, Choon SE, Marrakchi S, Burden AD, Tsai TF, Morita A, et al. Inhibition of the interleukin-36 pathway for the treatment of generalized pustular psoriasis. 2019;380:981–3. Copyright ^©^ (2019) Massachusetts Medical Society. Reprinted with permission from Massachusetts Medical Society.

### 2.1 Phase 1 open-label proof-of-concept trial

This phase 1 proof-of-concept trial (ClinicalTrials.gov ID, NCT02978690; study ID, 1368.11) was designed to investigate the safety, pharmacokinetics, pharmacogenomics, and efficacy of a single, open-label, IV dose of spesolimab at 10 mg/kg in patients with GPP flare (**Figure 1A**) (36). Adult patients were eligible for trial participation if they had a documented history of GPP, regardless of *IL-36RN* mutation status; presented with a GPP flare involving ≥10% of their body surface area; and had a GPPGA score ≥3 (i.e., moderate-to-severe intensity). Of 16 patients screened, seven patients at five trial sites received spesolimab on day 1 (baseline) and were followed up for 20 weeks thereafter (36). Patients received maintenance treatment with retinoids and/or methotrexate while they participated in the trial (36).

Of the seven patients, three had a homozygous *IL36RN* mutation (one of whom also had a heterozygous mutation in *CARD14* linked to pustular skin disease) and four had none of the target mutations (*IL36RN*, *CARD14*, and *AP1S3*) (36). All seven patients had adverse events (AEs) of mild or moderate severity after spesolimab infusion (36). Four patients (57.1%) had investigator-assessed drug-related AEs (upper respiratory tract infection and eosinophilia each occurred in two patients [28.6%]; urinary tract infection, arthralgia, chills, pain, vomiting, and infusion-related reaction each occurred in one patient [14.3%]) (36). There were no reports of severe or serious AEs (36). Laboratory parameters were normal in most patients following treatment with spesolimab (low hemoglobin, n = 2; elevated eosinophils, n = 2; elevated triglycerides, n = 2; elevated creatine kinase, n = 1; and low glucose, n = 1) (36).

**Figure 2:**
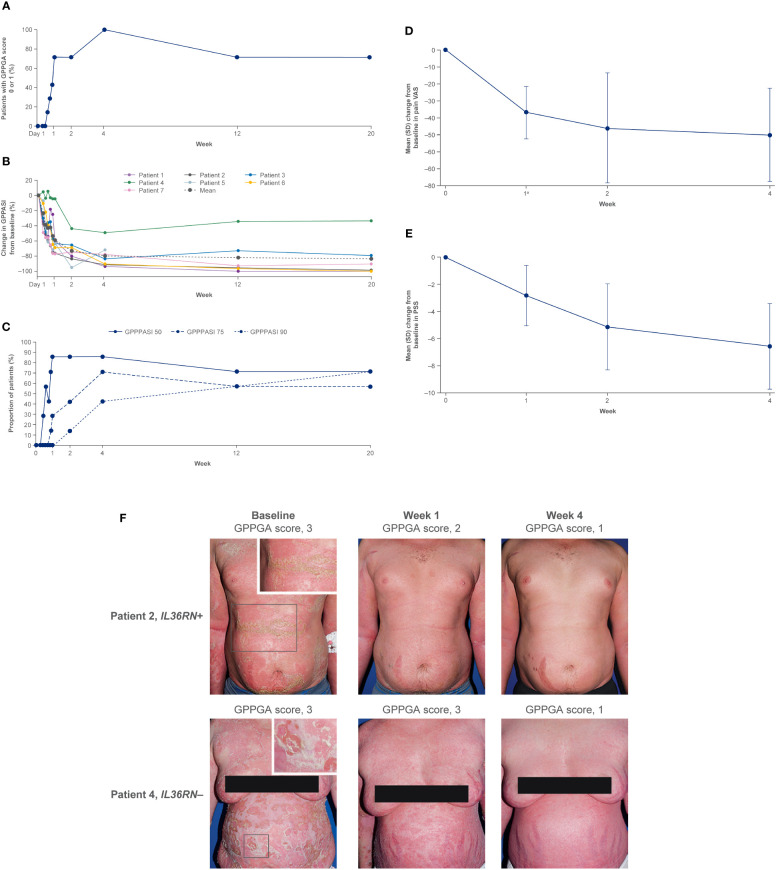
Proof-of-concept trial results (36). **(A)** Proportion of patients achieving a GPPGA score of 0 (clear) or 1 (almost clear). Data are based on the treated set (N = 7). At week 2, the GPPGA score for one patient was missing. **(B)** Change in GPPASI after spesolimab treatment. Data are based on the treated set (N = 7). One patient (patient 5) received methotrexate after week 4 for the treatment of pain, and data for this patient are censored at weeks 12 and 20. **(C)** Proportion of patients who achieved GPPASI 50/75/90 over time. The proportion of patients who achieved a decrease of more than 50%, 75%, or 90% in the GPPASI (GPPASI 50, 75, or 90) is shown over time. Analysis includes all patients with at least one available postbaseline value. One patient received methotrexate after week 4 for the treatment of pain; therefore, the data for weeks 12 and 20 are set to non-response. **(D)** Change from baseline in pain VAS through week 4. Mean (SD) change from baseline in pain VAS over time is shown. Analysis includes all patients with at least one available postbaseline value. ^a^ N = 6. **(E)** Change from baseline in PSS through week 4. Mean (SD) change from baseline in PSS over time is shown. Analysis includes all patients with at least one available postbaseline value. **(F)** Two study patients before and after treatment with spesolimab. Panel **(A)** shows photographs of two patients with generalized pustular psoriasis, one of whom had the *IL36RN* mutation (upper row) and one of whom did not have the mutation (bottom row). The images were taken at baseline (before treatment) and at weeks 1 and 4 after treatment with a single IV dose of spesolimab. GPPASI, Generalized Pustular Psoriasis Area and Severity Index; GPPASI 50/75/90, 50%/75%/90% or greater improvement in Generalized Pustular Psoriasis Area and Severity Index; GPPGA, Generalized Pustular Psoriasis Physician Global Assessment; IV, intravenous; PSS, Psoriasis Symptom Scale; SD, standard deviation; VAS, visual analog scale. From *N Engl J Med*. Bachelez H, Choon SE, Marrakchi S, Burden AD, Tsai TF, Morita A, et al. Inhibition of the interleukin-36 pathway for the treatment of generalized pustular psoriasis. 2019;380:981–3. Copyright ^©^ (2019) Massachusetts Medical Society. Reprinted with permission from Massachusetts Medical Society.

Efficacy data are presented in **Figure 2**. A GPPGA score of 0 or 1 (clear or almost clear skin) was attained in five of the seven patients (71%) by week 1 and in all patients by week 4 (36). The mean percent improvement in the GPPASI score from baseline was 59.0% at week 1, 73.2% at week 2, and 79.8% at week 4 (36). Pustules were completely cleared in three of the seven patients (43%) within 48 hours after treatment in five patients (71%) by week 1 and in six patients (86%) by week 2 (36). GPPGA, GPPASI, and pustule subscores were maintained up to week 20 (36). Improvements in PROs were evident from baseline to week 2 and were maintained to week 4 (36). The mean (standard deviation [SD]) change from baseline to week 2 in FACIT-F was 12.3 (10.1), in PSS was –5.14 (3.18), and in pain VAS was –45.9 (32.3) (36). Clinical images with corresponding GPPGA scores before and after spesolimab treatment are shown for two patients (**Figure 2F**) (36).

Biomarker findings supported the therapeutic targeting of IL-36R for the treatment of moderate-to-severe GPP (36, 43, 44). Spesolimab was associated with the rapid downregulation of biomarkers for IL-36–related pathways, neutrophilic activation and recruitment, T_h_1/T_h_17 and innate inflammation signaling, and keratinocyte-driven inflammation, all of which occurred as early as week 1 posttreatment (44). These reductions correlated with decreases in clinical disease severity (44). Additionally, a reduction in the mean (SD) level of C-reactive protein that approached normalization was observed from baseline to week 2 (from 69.4 [57.0] mg/dL to 4.5 [7.5] mg/dL) and was sustained until the last measurement was obtained at week 4 (36).

### 2.2 Phase 2 randomized, double-blind, placebo-controlled trial (Effisayil^™^ 1)

Effisayil^™^ 1 (NCT03782792; 1368.13) was the first multinational, randomized, double-blind, placebo-controlled trial in GPP flare and enrolled the largest group of patients with this condition (37, 38, 45). Adult patients were eligible for participation in the Effisayil^™^ 1 trial if they had a history of GPP consistent with the criteria for diagnosis according to the European Rare and Severe Psoriasis Expert Network criteria (14, 38). Patients had to have a GPP flare of moderate-to-severe intensity (defined as total GPPGA score ≥3, new or worsening pustules, a GPPGA pustulation subscore ≥2, and ≥5% body surface area with erythema and the presence of pustules). Although patients were enrolled regardless of *IL36RN* mutation status, DNA analyses of coding sequences for the three main GPP-associated genes (*IL36RN, CARD14*, and *AP1S3*) were performed. Recruitment was aided by the inclusion of a high number of centers located in Asia (20 of the 37 trial sites), which was consistent with the greater prevalence of GPP in this area (38).

Patients were randomized in a 2:1 ratio to receive a single IV dose of spesolimab 900 mg or placebo on day 1 (**Figure 1B**) (38). Patients from both treatment groups were eligible to receive open-label spesolimab (900 mg IV) on day 8 if they had persistent flare symptoms (based on a predefined threshold) and were followed to week 12 (38). Persistent flare symptoms were defined as follows: GPPGA total score ≥2 at the end of week 1 (range, 0 [clear skin] to 4 [severe disease]) and a clinician assessment of GPP severity based on a modified Physician Global Assessment and a GPPGA pustulation subscore ≥2 at week 1 (range, 0 [no visible pustules] to 4 [severe pustulation] (38). This led to cross-over from placebo to spesolimab for some patients (38). Escape medication (the treating physician’s choice of standard of care) could be given to a patient if GPP severity and progression worsened within the first week after randomization (37). Any patient who received escape medication was considered a non-responder in the analysis for the primary and key secondary evaluation at week 1 (38). After week 1, one rescue open-label dose of spesolimab 900 mg IV could be administered to a patient with GPP flare recurrence who had previously achieved a clinical response (37). Patients with worsening disease but who did not achieve a clinical response could be given an escape treatment chosen by the treating physician (37). The primary endpoint was a GPPGA pustulation subscore of 0 (clear) at the end of week 1 (38). The key secondary endpoint was a GPPGA total score of 0 or 1 (clear or almost clear) at the end of week 1 (38). Secondary endpoints were assessed at week 4 and included ≥75% improvement in GPPASI (GPPASI 75) and PROs (DLQI, FACIT-F, PSS, and pain VAS) (38). Additional endpoints included assessment of anti-drug antibodies and exploration of biomarkers in GPP flare (37).

A total of 53 patients were enrolled and randomized to receive treatment with spesolimab (N = 35) or placebo (N = 18) (38). Baseline demographic and clinical characteristics differed between the spesolimab and placebo groups regarding female sex (21/35 [60%] and 15/18 [83%], respectively), Asian race (16/35 [46%] and 13/18 [72%], respectively), and median GPPASI score (27.4 [interquartile range (IQR), 15.5–36.8] and 20.9 [IQR, 12.0–32.0], respectively) (38). A baseline GPPGA pustulation subscore of 3 was reported in 16/35 (46%) and 7/18 (39%) patients and a subscore of 4 was reported in 13/35 (37%) and 6/18 (33%) of patients in the spesolimab and placebo groups, respectively (38). A total of seven patients (spesolimab group, 5/35 [14%]; placebo group, 2/18 [11%]) had mutations in *IL36RN* (38). Escape medication was given to two patients in the spesolimab group and one patient in the placebo group during week 1, and to four patients in the spesolimab group and four patients in the placebo group after week 1. At day 8, 12/35 patients (34.3%) in the spesolimab group and 15/18 patients (83.3%) in the placebo group received a single open-label dose of spesolimab (38). After day 8, four patients in the spesolimab group and two patients in the placebo group required rescue treatment with spesolimab (38). After 12 weeks of treatment, 39 patients were enrolled on the open-label extension trial, Effisayil^™^ ON.

**Figure 3:**
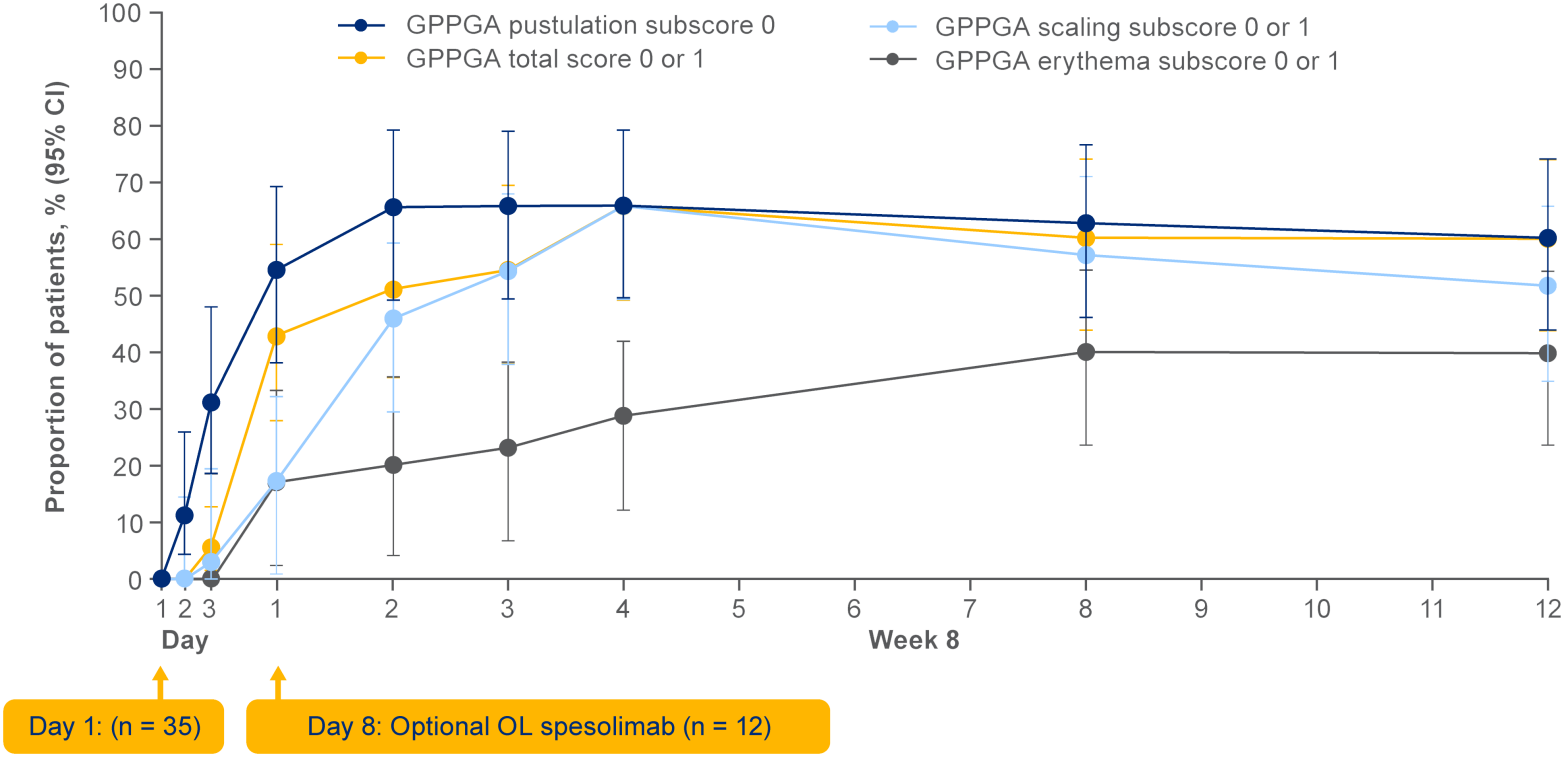
Effisayil^™^ 1 trial results (46). Proportion of patients randomized to spesolimab with a GPPGA pustulation subscore of 0 or GPPGA total, scaling, or erythema scores of 0 or 1 through week 12. Treatment effect in patients initially randomized to spesolimab who received up to two doses of spesolimab: day 1 (n = 35) and optional dose at day 8 (n = 12). Missing values, any use of other medications for GPP, or use of spesolimab for the treatment of a new GPP flare were regarded as non-response for this analysis. *Arrowheads* indicate the days of intravenous spesolimab administration. GPPGA, Generalized Pustular Psoriasis Physician Global Assessment; OL, open label. Reprinted from *J Am Acad Dermatol*, 2023;89;36–44, Elewski B, Lebwohl MG, Anadkat MJ, Barker J, Ghoreschi K, Imafuku S, et al. Rapid and sustained improvements in Generalized Pustular Psoriasis Physician. Global Assessment scores with spesolimab for treatment of generalized pustular psoriasis flares in the randomized, placebo-controlled Effisayil 1 study, with permission from the American Academy of Dermatology, Inc. Published by Elsevier. All rights reserved.

**Figure 4:**
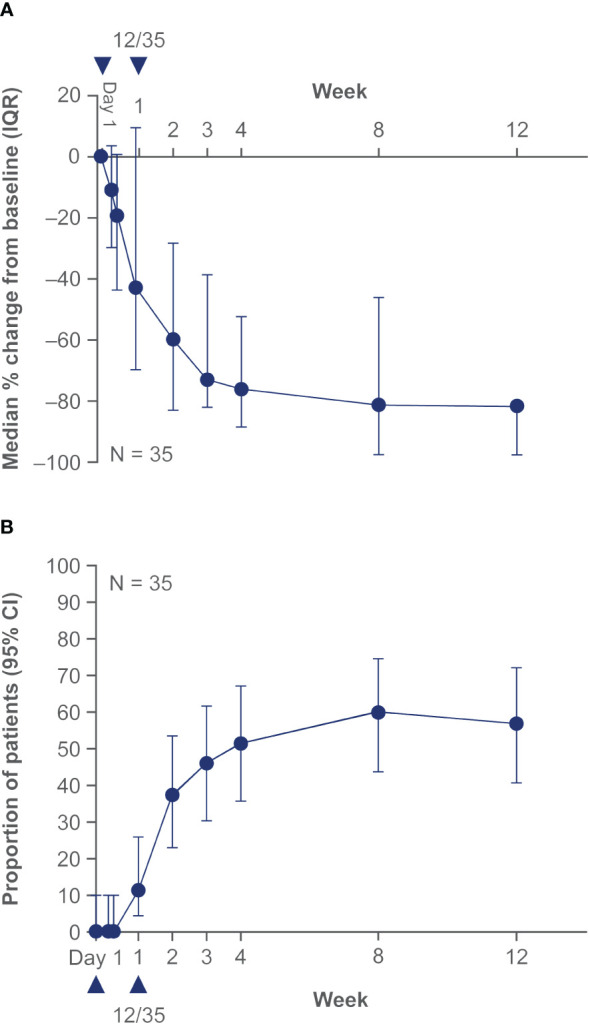
Effisayil^™^ 1 trial results (38). Treatment response in patients who received up to two doses of spesolimab at day 1 and optional dose at day 8 in spesolimab arm. **(A)** GPPASI and **(B)** GPPASI 75: The dataset includes observed cases in patients randomized to spesolimab who received up to two doses of spesolimab, including patients who received OL spesolimab at day 8. The arrowhead indicates the days of IV spesolimab administration. For this analysis, any values post OL spesolimab at day 8 are used; any values post use of escape medication or rescue medication with spesolimab are imputed as the worst outcome in the calculation of median and quartiles. CI, confidence interval; GPPASI, Generalized Pustular Psoriasis Area and Severity Index; GPPASI 75, 75% or greater improvement in Generalized Pustular Psoriasis Area and Severity Index; IQR, interquartile range; From *N Engl J Med*. Bachelez H, Choon SE, Marrakchi S, Burden AD, Tsai TF, Morita A, et al. Trial of spesolimab for generalized pustular psoriasis. 2021;385:2431–40. Copyright ^©^ (2021) Massachusetts Medical Society. Reprinted with permission from Massachusetts Medical Society.

**Figure 5:**
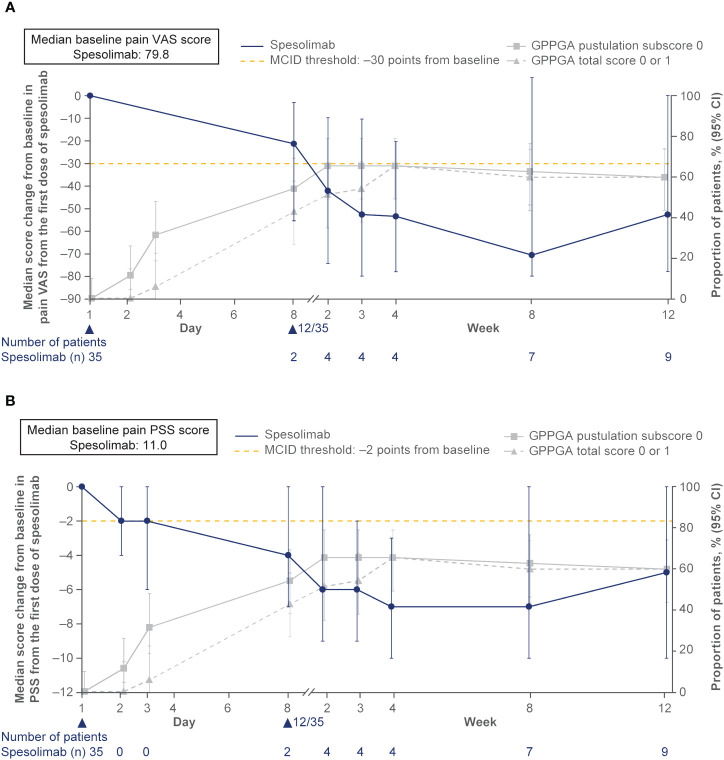
Effisayil^™^ 1 trial results (47). **(A)** Pain VAS and **(B)** PSS: Absolute change from baseline in PRO scores over time in patients randomized to receive spesolimab on day 1. Treatment effect in patients who received up to two doses of spesolimab: day 1 (N = 35) and optional dose at day 8 (n = 12). The arrowhead indicates the days of IV spesolimab administration. Any use of other medication for GPP or use of spesolimab for the treatment of a new GPP flare were regarded as non-response for this analysis. The gray lines show the proportion of patients who achieved a GPPGA pustulation subscore of 0 and GPPGA total score of 0 or 1 over time. The dashed lines indicate PRO MCID threshold of 30 points for pain VAS and two points for PSS (Rentz, et al. Reliability, validity, and the ability to detect change of the psoriasis symptom scale (PSS) in patients with plaque psoriasis. *J Dermatolog Treat* (2020) 31:460–9; Lee, et al. Clinically important change in the visual analog scale after adequate pain control. *Acad Emerg Med.* (2003) 10:1128–30.) CI, confidence interval, GPPGA, Generalized Pustular Psoriasis Physician Global Assessment; MCID, minimal clinically important difference; PSS, Psoriasis Symptom Scale; VAS, visual analog scale. Navarini AA, Prinz JC, Morita A, Tsai TF, Viguier MA, Li L, et al. Spesolimab improves patient-reported outcomes in patients with generalized pustular psoriasis: Results from the Effisayil 1 study. *J Eur Acad Dermatol Venereol.* 2023;37(4):730–6. Copyright ^©^ 2022 The Authors. Reproduced with permission of John Wiley & Sons Inc.

**Figure 6:**
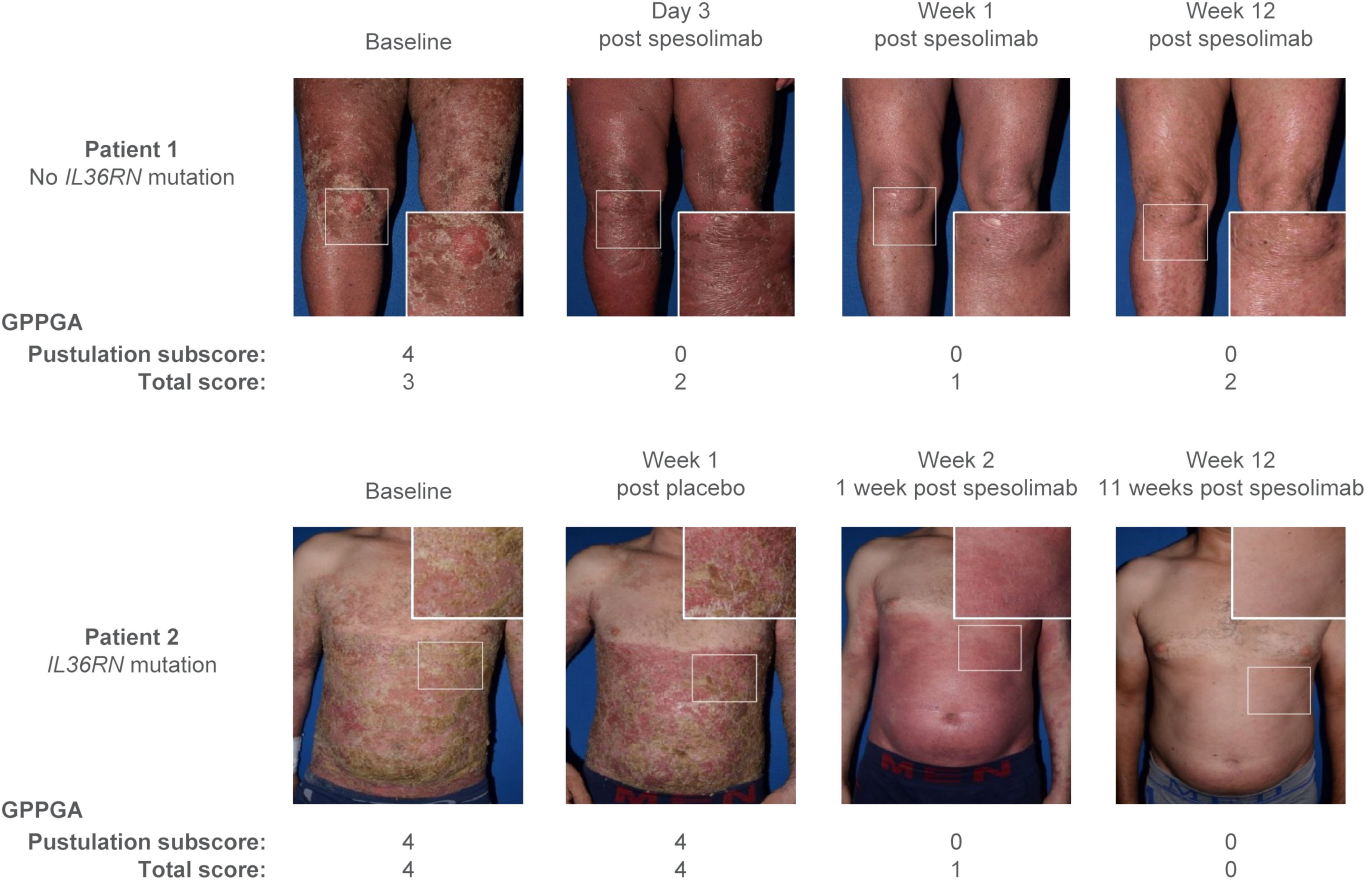
Effisayil^™^ 1 trial results (46). Two study patients before and after treatment with spesolimab. Patient 1 was initially randomized to spesolimab; patient 2 was initially randomized to placebo and received an OL dose of spesolimab on day 8. GPPGA, Generalized Pustular Psoriasis Physician Global Assessment; *IL36RN*, interleukin-36 receptor antagonist; OL, open label. Parkinson, James (2023), Elewski et al. **Supplementary Data**, Mendeley Data, V1, doi: 10.17632/nz35b7b26d.1, data.mendeley.com/datasets/nz35b7b26d/1, is licensed under CC BY 4.0.

Efficacy data are presented in **Figures 3**–**5**. At the end of week 1 (i.e., day 8), a GPPGA pustulation subscore of 0 (no visible pustules) was achieved in 19/35 (54%) patients receiving spesolimab versus 1/18 (6%) receiving placebo, and a GPPGA total score of 0 or 1 (clear or almost clear skin) was achieved by 15/35 (43%) patients in the spesolimab group and 2/18 (11%) patients in the placebo group (38). At week 12, 21/35 (60%) patients randomized to spesolimab achieved a GPPGA pustulation subscore of 0, and 21/35 (60%) achieved a GPPGA total score of 0 or 1 (46). Median reduction from baseline in GPPASI was approximately 80% at week 4 and was sustained to week 12 (38). A total of 18/35 (51%) patients receiving spesolimab achieved a GPPASI 75 at week 4, which was sustained to week 12 when 20/35 (57%) patients achieved GPPASI 75 (38). Patients who received spesolimab demonstrated improvements from baseline in all four PROs within 1 week of treatment (median [quartile (Q)1, Q3]; pain VAS, –21.3 [–55.3, –3.1], FACIT-F, 7.0 [1.0, 20.0]), DLQI, –2.5 [–8.0, 1.0], and PSS, −4.0 [−7.0, 0.0]), which were sustained over 12 weeks (47). These improvements corresponded to the achievement of MCIDs at week 1 that were also sustained over 12 weeks (47). Patients in the placebo group experienced improvements in PROs and achievement of MCIDs after receipt of open-label spesolimab at week 1 (47). Clinical images with corresponding GPPGA scores before and after spesolimab treatment are shown for two patients (**Figure 6**) (46). A *post hoc* sensitivity analysis of the primary and key secondary endpoints was undertaken to adjust for baseline imbalances in sex, race, and GPPASI scores and produced results consistent with the primary analysis (38).

At week 1, AEs occurred in 23/35 (66%) patients in the spesolimab group and in 10/18 (56%) patients in the placebo group (38). Infections at week 1 were reported in 6/35 (17%) patients receiving spesolimab and in 1/18 (6%) patients receiving placebo (38). Serious AEs at week 1 were reported in 2/35 (6%) patients receiving spesolimab, but were not reported in any of the patients receiving placebo (38). At week 12, AEs occurred in 42/51 (82%) patients who had received at least one dose of spesolimab (including those initially randomized to placebo who received open-label spesolimab at day 8), and serious AEs were reported in 6/51 (12%) patients (38). Infections at week 12 were reported in 24/51 (47%) patients, including three cases each of urinary tract infection and influenza and two cases each of folliculitis, otitis externa, upper respiratory tract infection, and pustule (38). [Pustular psoriasis was excluded as an AE from the published safety analysis (38)]. Symptoms observed in two patients receiving spesolimab were reported as drug reaction with eosinophilia and systemic symptoms (DRESS) with European Registry of Severe Cutaneous Adverse Reactions (categories: “no”, “possible”, “probable”, or “definite” DRESS) score indicating case 1 as “no DRESS”, and case 2 as “possible DRESS” (38, 48). In case 1, the patient received concomitant medications that could be associated with DRESS (cefuroxime, cefepime, and paracetamol), and furthermore later reported a history of adverse drug reactions to cephalosporins (in particular cefuroxime). In case 2, the patient received spiramycin, and the event reoccurred after spiramycin rechallenge (details are provided in the publication **Supplementary Appendix**) (38).

Anti-drug antibodies were detected in 23/50 (46%) patients who received at least one dose of spesolimab, with a median time to detection of 2.3 weeks after spesolimab administration (38). Exploration of biomarkers identified more than 5200 differentially expressed gene transcripts in biopsies from GPP skin lesions versus non-lesional skin, including genes associated with IL-36, neutrophil recruitment, proinflammatory cytokines, and skin inflammation (49). Transcriptional modulation of a significant number of genes was observed 1 week after spesolimab administration and remained at 8 weeks posttreatment. In GPP lesional skin biopsies, significant decreases in the expression of genes associated with proinflammatory mediators, neutrophil recruitment, keratinocyte-mediated inflammation and proliferation, and IL-36 ligands were observed at week 8 posttreatment versus baseline (49). Importantly, significant changes in differential gene expression were demonstrated in patients who achieved the primary endpoint of GPPGA pustulation subscore of 0 at week 1 (49).

A subgroup analysis of the 29 Asian participants in Effisayil^™^ 1 demonstrated comparable efficacy and safety to that in the overall trial population (50). A GPPGA pustulation subscore of 0 at week 1 occurred in 10/16 (63%) patients in the spesolimab group and 1/13 (8%) patient in the placebo group (risk difference, 54.8; 95% confidence interval [CI] 17.3 to 79.8), and a GPPGA total score of 0 or 1 was achieved by 8/16 (50%) and 2/18 (15%) patients, respectively (risk difference, 34.6; 95% CI –3.1 to 64.7) (50). At least one AE was reported in 11/16 (69%) patients treated with spesolimab and 8/13 (62%) patients who received placebo (50). For patients randomized to receive spesolimab, continuous improvement in PROs was observed over 8 weeks, with some plateauing between weeks 8 and 12; furthermore, MCIDs were achieved for all four PRO scales and were sustained to week 12 (50). Also, markers of systemic inflammation were normalized in spesolimab-treated patients (50). Efficacy was also consistent for the trial duration across all other prespecified Effisayil^™^ 1 subgroups (sex, age, race, body mass index, GPPGA pustulation subscore at baseline, GPPGA total score at baseline, Japanese Dermatological Association GPP severity score at baseline, presence of plaque psoriasis at baseline, and *IL36RN* status) (51).

(A summary of the main efficacy and safety data from these two clinical trials are also presented in a **Supplementary Video**).

## 3 Discussion

Data from the proof-of-concept and Effisayil^™^ 1 trials demonstrate the efficacy and safety of spesolimab and support its use in the treatment of patients with acute GPP flares (36, 38). Patients treated with spesolimab had a significantly higher rate of pustule clearance and skin improvement at 1 week postdose versus those who received placebo, and the effect was sustained through the 12-week trial duration of Effisayil^™^ 1 (38). The overall safety and tolerability profile of spesolimab was favorable, although it was associated with infections and systemic reactions (38), and was consistent with other biologic agents (52). PRO data indicated that the effect of spesolimab treatment translated into a better quality of life for the patient via significant improvement in pain, fatigue, and overall skin condition (38). Biomarker data provided additional support for the intended spesolimab mechanism of action and confirmed that its clinical efficacy was associated with modulation of critical pathways linked to IL-36 in GPP pathogenesis (44, 49). These clinical trials of spesolimab also facilitated the development of the clinically relevant GPP-specific endpoints GPPGA and GPPASI. GPPGA and GPPASI scoring for measuring skin symptom severity in patients with GPP were recently shown to be valid, reliable, and sensitive; thus, supporting the use of these instruments as suitable endpoints in future GPP clinical trials and confirming their potential use as standard clinical tools for the assessment of disease severity (40). The use of PRO questionnaires in these GPP trials allowed the evaluation of treatment efficacy from the patient’s perspective. Recent real-world evidence demonstrated the detrimental impact of GPP on an individual’s quality of life and suggested that previous therapies used to treat GPP had not adequately addressed this issue (4, 53). Further studies have investigated the burden of disease (clinical, humanistic, and economic) in patients living with GPP (2, 3, 5, 54, 55).

Prior to the first regulatory approval of spesolimab in 2022, no GPP-specific treatments were approved in the US or Europe. Non-biologic systemic agents, such as methotrexate, retinoids (acitretin), and cyclosporine, were used to treat GPP flares; however, methotrexate has a slow onset of action, and adverse effects/toxicities limit the use of retinoids and cyclosporine (6). Several biologics are approved in Japan for the treatment of GPP; including, anti-tumor necrosis factor (TNF)-α agents (adalimumab, infliximab, and certolizumab pegol), anti-interleukin (IL)-17 agents (IL-17A: secukinumab and ixekizumab; IL-17 receptor: brodalumab), and anti-IL-23 agents (risankizumab and guselkumab); the supporting efficacy data mainly arise from small, uncontrolled clinical trials (6, 7, 56). In other countries, some of these agents have been used off-label to treat GPP (6). No data are available to compare efficacy outcomes in patients with GPP who were treated with spesolimab versus other agents. Recently, data were reported for a second anti–IL-36 receptor monoclonal antibody, imsidolimab (AnaptysBio, Inc, San Diego, CA, USA) (57), from a 30-week phase 2, open-label, single-arm clinical trial of eight patients with GPP, in which 6/8 (75%) of patients responded to treatment (measured via the Clinical Global Impression scale) at weeks 4 and 16 (58).

GPP presents various challenges when conducting a randomized placebo-controlled trial; it is a rare disease, episodic and unpredictable in nature, with sudden and self-limiting occurrence of acute flares. The potential for clinically severe disease renders the extended use of placebo unethical. Thus, the 1-week randomization period, after which 34% of patients in the spesolimab group and 83% of patients in the placebo group received open-label spesolimab, restricted the use of conventional analyses to compare the two treatment groups after week 1 (38). Nonetheless, Effisayil^™^ 1 remains the largest and only randomized clinical trial to date in patients experiencing GPP flares. The 12-week follow-up period provided valuable information on the longer-term efficacy and safety of spesolimab, although definitive long-term data will come from an open-label extension study (Effisayil^™^ ON) several years hence.

Regarding other clinical trials with spesolimab, pharmacokinetic and safety data from phase 1 clinical trials in healthy volunteers were used to inform spesolimab dose selection in patients with GPP in the subsequent proof-of-concept and Effisayil^™^ 1 trials (59). Effisayil^™^ 2 and Effisayil^™^ ON are additional clinical trials to investigate the efficacy and safety of spesolimab in patients with a history of GPP. Effisayil^™^ 2 (NCT04399837, 1368–0027; N = 123) was a 48-week dose-finding trial of spesolimab for flare prevention in patients with GPP whose skin is clear or almost clear at trial entry (60). The trial was completed in December 2022 (61), and high-dose spesolimab was superior to placebo in preventing flares, with an 84% reduction in the risk of flare development (HR [95% CI] 0.16 [0.05, 0.54]; P = 0.0005), and no flares occurring in the high-dose spesolimab group after week 4 (62). Effisayil^™^ ON (NCT03886246; 1368–0025; N = 131) is an ongoing open-label extension study to investigate the long-term safety and efficacy of spesolimab in patients who took part in Effisayil^™^ 1 or Effisayil^™^ 2 (63). Additional clinical trials of spesolimab in GPP include phase 3 expanded access trials in Japan (NCT05200247; completed 2023) and China (NCT05239039; completed 2023); a post-marketing surveillance study of GPP with acute symptoms in Japan (NCT05670821; recruiting); and a phase 4 multi-center, open-label, post-marketing trial of treatment for repeated flares (NCT06013969; Effisayil^®^ REP; recruiting). Data from clinical trials are supported by the first published real-world case of spesolimab treatment in GPP acute disease flare, in which complete and rapid response to spesolimab therapy was reported in a 60-year-old woman who presented to the emergency department (64). The patient experienced total resolution of symptoms within 40 hours of receiving spesolimab via IV infusion; no AEs were reported, and the patient was discharged 2 days posttreatment. This case demonstrates the potential of spesolimab to improve morbidity and mortality in patients with GPP and reduce the length of inpatient stay (64). Although no comparator was present in this case report, a large retrospective review of patients with acute GPP (N = 102) reported that the duration of admission and duration of pustular flare was numerically larger (admission: mean, 10.3 days; range, 3–44 days; pustular flare: mean, 16 days; range, 7–60 days) (2). Several other case reports and case series have since been published that also report the successful treatment of patients with GPP using spesolimab (65–72). Further data describing the acute and long-term burden of GPP in various populations have also been published (73–78). Lastly, a global Delphi consensus describing clinically meaningful goals for GPP diagnosis, treatment, and assessment was published in 2023 (79), as was a consensus statement from the National Psoriasis Foundation, which advocated timely access to FDA-approved therapies for GPP, such as spesolimab, to reduce the risk of mortality in affected patients (80). In addition to GPP, spesolimab is being investigated in other diseases; phase 2 clinical trials of spesolimab in the treatment of hidradenitis suppurativa are active (NCT04876391), recruiting (NCT05819398), or recently completed (NCT04762277); a phase 2 clinical trial of spesolimab in the treatment of pyoderma gangrenosum is recruiting (NCT06092216); and a phase 2/3 clinical trial of spesolimab in the treatment of Netherton syndrome is underway (NCT05856526).

In conclusion, spesolimab is an IL-36 receptor antagonist, approved for the treatment of GPP in patients with and without flares. The proof-of-concept and Effisayil^™^ 1 trials were the first clinical trials that demonstrated the efficacy and safety of spesolimab in providing rapid and sustained clinical improvement for patients with GPP flares, which translates into increased quality of life, by offering a targeted therapy for GPP.

## Author contributions

EG: Writing – original draft, Writing – review & editing. AN: Writing – original draft, Writing – review & editing.
