# Supplementary material for: Spesolimab for generalized pustular psoriasis: a review of two key clinical trials supporting initial US regulatory approval
Source: Front Immunol. 2024 Jul 22;15:1359481. doi: 10.3389/fimmu.2024.1359481 (PMC11298804; doi:10.3389/fimmu.2024.1359481)
Supplement: Supplementary file 1 [file DataSheet_1.pdf]

Spesolimab Treatment for People with Generalized Pustular Psoriasis: A Summary of Two Key Clinical Studies

This plain language summary reports information from an article on two key clinical studies of a medicine called spesolimab that is used to treat **generalized pustular psoriasis** (or **GPP** for short).

What is GPP?

- GPP is a rare skin condition.
- People with GPP have **flares** of disease when the skin becomes suddenly red and sore, and painful pus-filled blisters (called pustules) appear on the body.
- GPP can affect other parts of the body, as shown in the figure below. People with GPP may sometimes need treatment in the hospital

People with GPP have skin problems and may have symptoms affecting other parts of the body

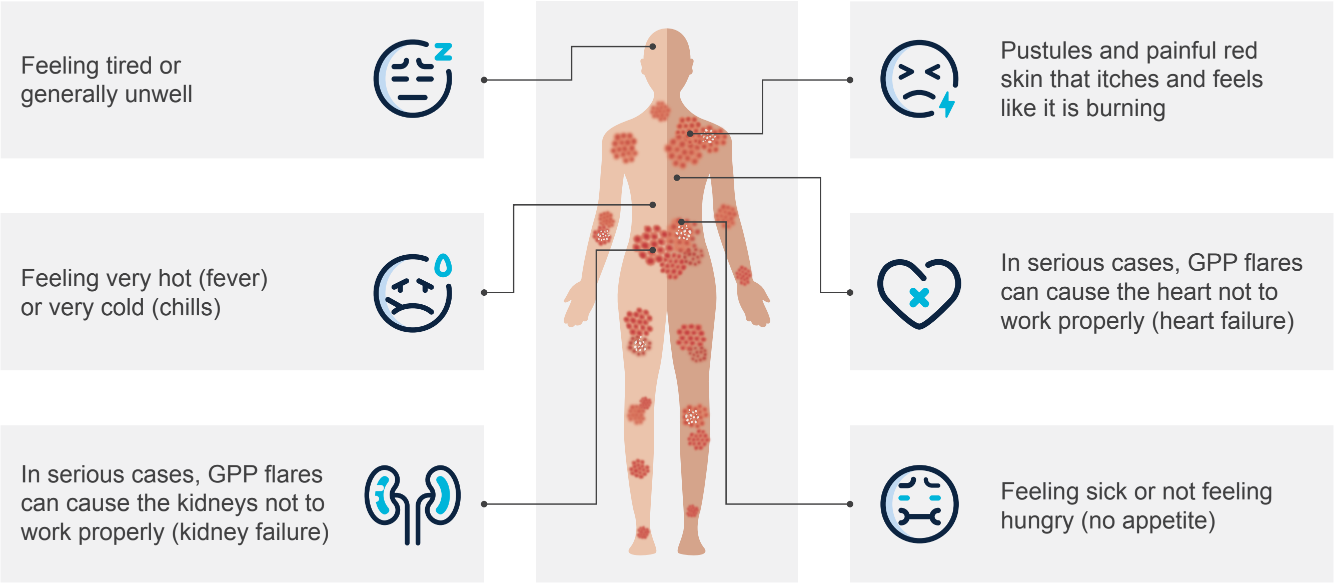

- The exact cause of GPP has not been found yet. Several factors can trigger flares, including stress, infections of the throat or airways, and taking certain types of medicine.
- In people with GPP, a **trigger** makes the immune system think there is a threat to the body, and an **immune reaction** occurs in which various chemicals are produced.
- One of these chemicals is called **interleukin-36** (or **IL-36** for short). In people with GPP, IL-36 does not work properly and does not “turn off”, so the immune reaction becomes over-active.
- This causes redness, swelling, and pain in the skin (called inflammation) and also causes white blood cells to collect in the skin.
- The white blood cells release more chemicals that damage the skin cells. When the white blood cells die, they form pus.

What is spesolimab and how does it work?

- **Spesolimab** is a new medicine that blocks the effects of IL-36 in people with GPP.
  - This stops the immune system from becoming over-active and prevents the release of the chemical messages that cause inflammation, as shown in the figure below.
- Spesolimab stops a GPP flare or makes it less severe.
- Spesolimab is given via a needle inserted into a vein in the arm (called an infusion).

The immune system "over reacts" in people with GPP, due to a faulty IL-36 pathway – spesolimab helps to correct this

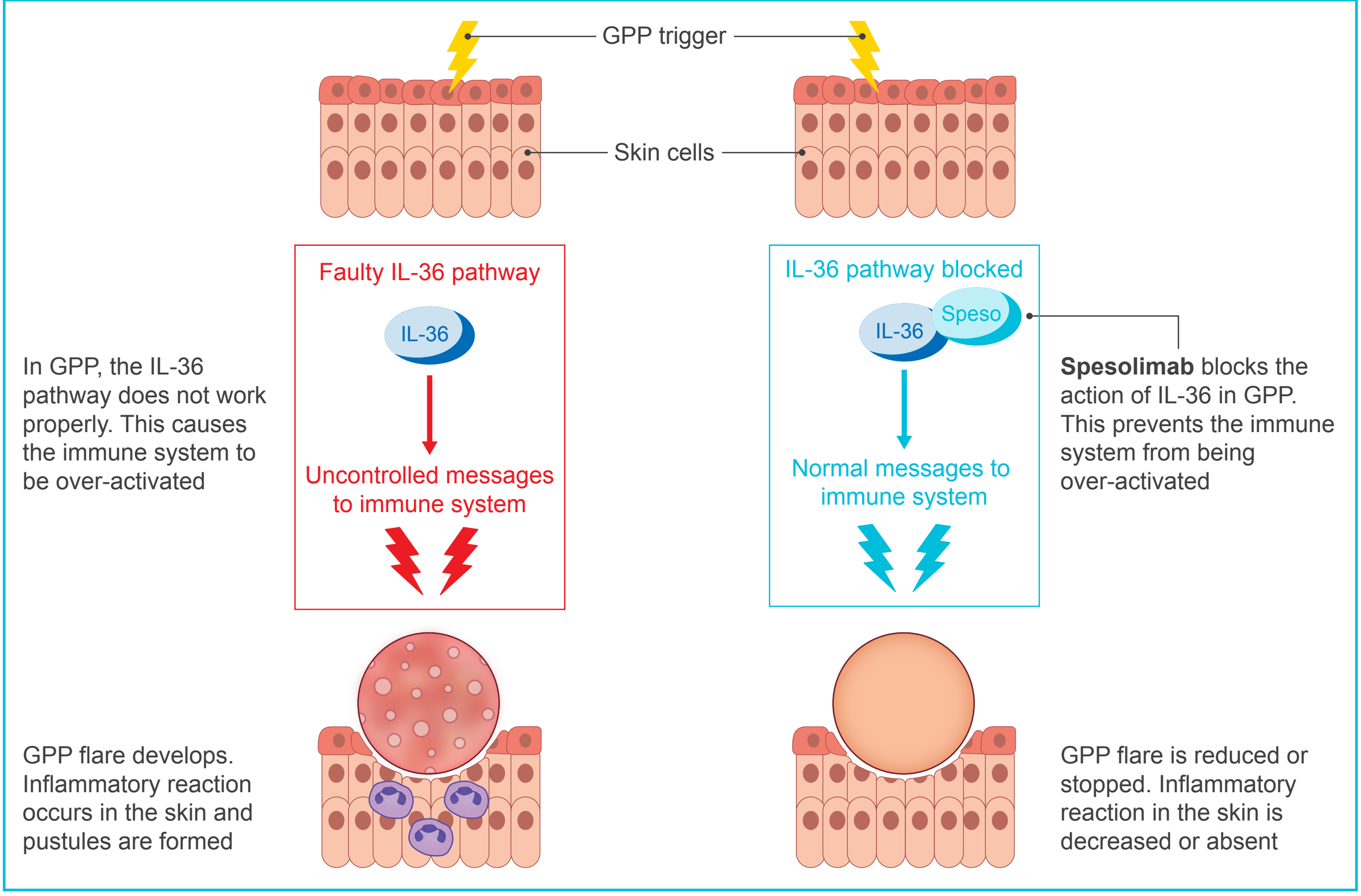

Speso = spesolimab

- Spesolimab was initially approved for use in the United States (US), Japan, and Europe in 2022.
  - This means that doctors in those countries can prescribe spesolimab to people with GPP.
- The initial approval in the US was based on the results of two key clinical studies.
  - These were the **phase 1 study** and the **phase 2 study** (also called **Effisayil™ 1**).

What did the phase 1 spesolimab clinical study look at?

- This phase 1 study was done to learn if spesolimab treatment had any side effects.
- Researchers also wanted to find out if treatment with spesolimab did the following:
  - Reduced the amount of skin that was affected by GPP.
  - Reduced how severe the disease was.
  - Led to a complete or almost complete clear-up of GPP skin lesions (meaning pustules, skin redness, and skin flaking or peeling).
- Seven people who were having GPP flares received spesolimab on the first day of the study (day 1). They visited the study doctors to assess their skin at regular intervals over the next 20 weeks.

What were the main results of the phase 1 spesolimab clinical study?

- All seven people in the study had side effects after taking spesolimab medicine. Of these, four people had side effects that the study doctors thought were related to taking spesolimab.
  - All of the side effects were graded as mild or moderate.
- The other results of the study are shown in the figure below.

Phase 1 study main results

After 1 week of the study

GPP skin lesions had **gone away or almost gone away** in **5 of the 7 (71%)** people who received **spesolimab**

Spesolimab

GPP skin lesions gone/almost gone?

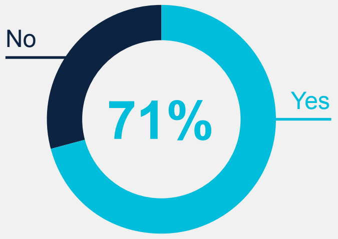

After 4 weeks of the study

GPP skin lesions had **gone away or almost gone away** in **all 7 (100%)** people who received **spesolimab**

GPP skin lesions gone/almost gone?

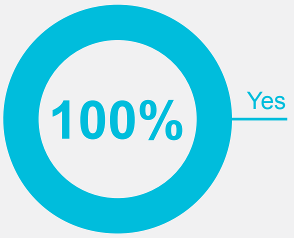

After 4 weeks of the study

The **amount of skin affected** by GPP and the **disease severity** improved by almost **80%**

Less skin affected by GPP and skin disease less severe?

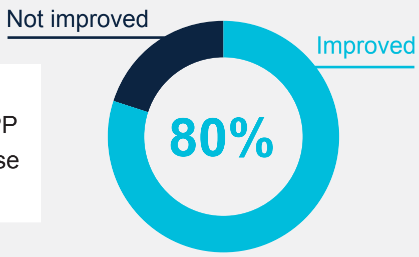

These effects on the skin lasted to the end of the study (20 weeks)

What do the results of the phase 1 spesolimab clinical study mean?

- This study showed that in people with a GPP flare, one dose of spesolimab did not cause any serious side effects.
- Spesolimab cleared up GPP skin lesions quickly, and the effects continued throughout the study.

What did the phase 2 spesolimab clinical study (Effisayil™ 1) look at?

- The phase 2 study (Effisayil™ 1) was done to find out if spesolimab treatment led to a complete clear-up of GPP skin pustules.
- This study also looked at whether spesolimab treatment led to a complete or almost complete clear-up of all GPP skin lesions.
- People taking part in this study were asked to report how GPP affected their health and well-being (called quality of life) during the study.
- In addition, the researchers looked at whether spesolimab treatment had any side effects.
- In total, 53 people who were experiencing GPP flares took part in this 12-week study. The study details are shown in the figure below.

Phase 2 study (Effisayil™ 1) of spesolimab in people with GPP flares

53 people aged 18 – 75 years and who were experiencing a GPP flare took part in the study

The 53 people were then divided into two groups at random

Day 1 (study starts)

35 people were given one dose of **spesolimab***	18 people were given a non-active medicine called a **placebo***

(*The people in the study and the study doctors did not know which medicine was given)

Each person was then checked regularly by the study doctors over the next 12 weeks to see if they had any side effects from spesolimab, and to see and if spesolimab had any effect on the GPP flare

Day 8

Each person was checked by the study doctors after 1 week

If a person kept having GPP flare symptoms, they were allowed to have a dose of spesolimab. If this happened, the person and their doctor knew they had been given spesolimab (rather than placebo)

12 of the 35 people in the **spesolimab group** were given another dose of spesolimab	15 of the 18 people in the **placebo group** were given a dose of spesolimab

Day 8 to week 12

People were allowed to have another dose of spesolimab if they had a new GPP flare later on in the study

Week 12 (study stops)

49 people were checked by study doctors until the end of the study. People who finished the study could then take part in a 5-year study of spesolimab, and 39 people chose to do this

(After 12 weeks, 51 people taking part in the study had received at least one dose of spesolimab.)

What were the main results of the phase 2 spesolimab clinical study (Effisayil™ 1)?

- The main results of the study are shown in the figure below.

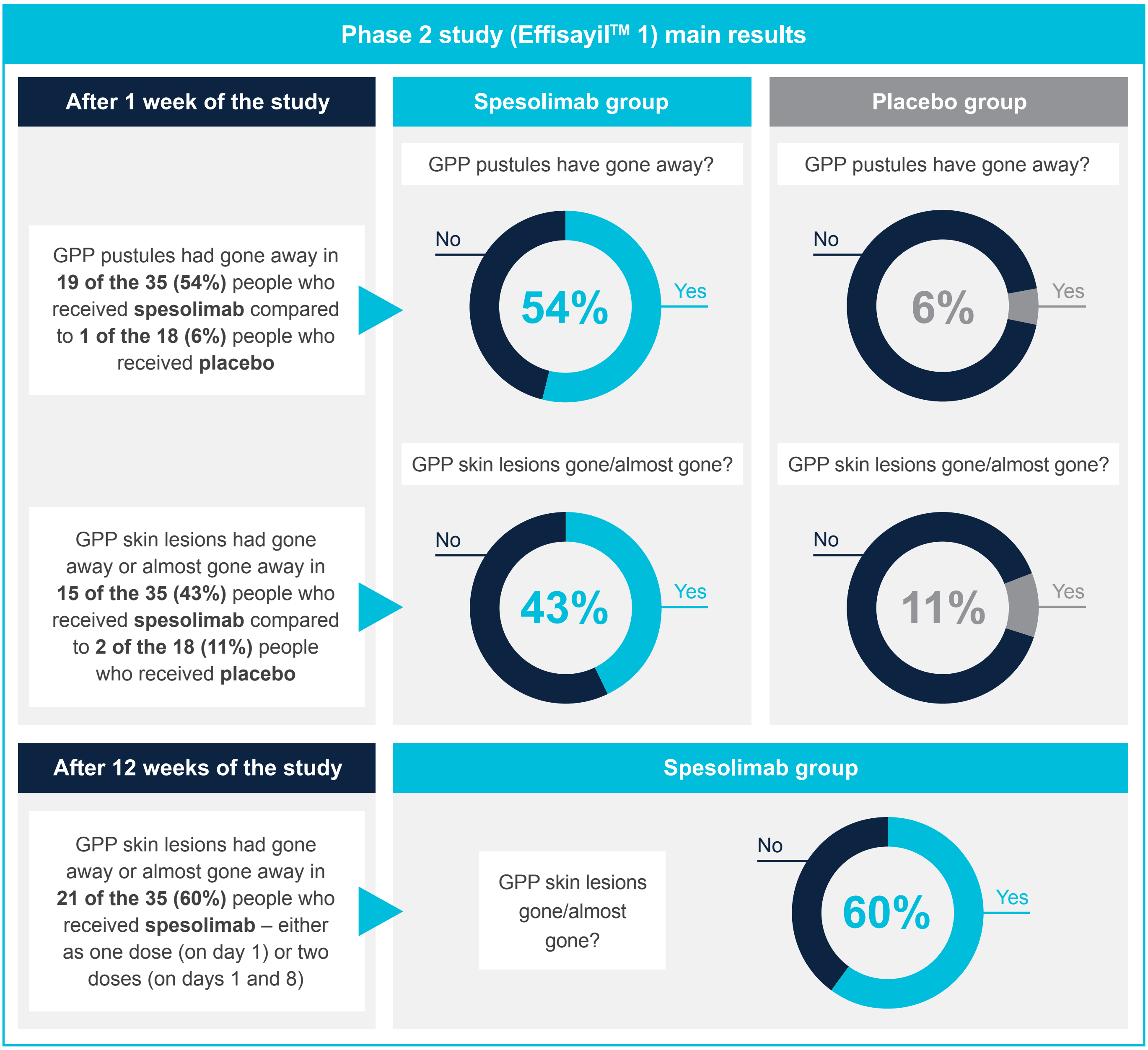

- People reported that their health and well-being (quality of life) improved after taking spesolimab. They had less pain, itching, redness, burning of the skin, and felt less tired.
- Some people experienced adverse events after taking their medicine. Most of the adverse events were not serious (meaning the adverse event did not need treatment in the hospital and did not cause death or disability). (An "adverse event" is an unintended response that may or may not have been caused by the medicine taken; whereas a "side effect" is caused by the medicine taken.)
- After 1 week of the study:
  - 23 of the 35 (66%) people who received spesolimab had adverse events compared with 10 of the 18 (56%) people who received placebo.
  - Common adverse events included fever and feeling dizzy.
    - Fever occurred 2 of 35 (6%) people who received spesolimab and in 4 of 18 (22%) people who received placebo; feeling dizzy occurred in none of 35 (0%) people who received spesolimab and in 2 of 18 (11%) people who received placebo.

- After 12 weeks of the study:
  - 6 of 51 (12%) people who received one or more doses of spesolimab had experienced serious adverse events, as shown in the figure below.

1 case of each of the following:

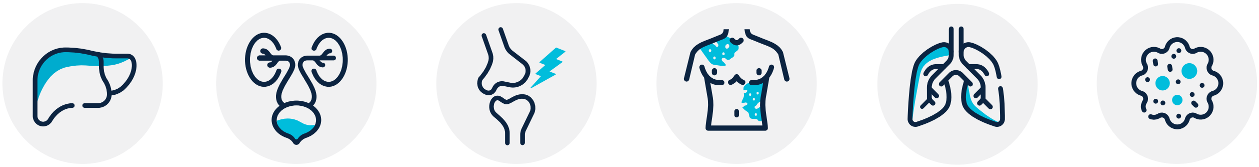

Possible drug-related liver injury | Urinary tract infection | Arthritis | Psoriasis | Influenza | Skin cancer

The potential drug-induced liver injury occurred at the same time as the GPP flare

2 cases of a drug reaction called DRESS (which stands for drug reaction with eosinophilia and systemic symptoms)

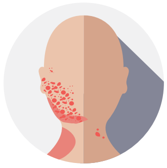

Some of the symptoms of DRESS are like those of a GPP flare. The participants were taking other medicines at the time this side effect occurred

What do the results of the phase 2 spesolimab clinical study (Effisayil™ 1) mean?

- This study showed that in people with a GPP flare, one dose of spesolimab was better than placebo at clearing up pustules and skin lesions after 1 week.
- The improvements in skin lesions continued for up to 12 weeks.
- Spesolimab also reduced pain, itching, burning, tiredness, and improved the overall well-being (quality of life) of the people who took the drug during the study
- Adverse events with spesolimab were broadly similar to those with placebo after 1 week of the study.

How to say:

Interleukin (in-ter-loo-kin)

Psoriasis (sore-eye-a-sis)

Pustular (puhs-choo-lar)

Spesolimab (spez-oh-lee-mab)

Where can I find further information?

You can find more information on GPP here:

- National Psoriasis Foundation
  https://www.psoriasis.org/pustular/
- American Academy of Dermatology
  https://www.aad.org/public/diseases/psoriasis/treatment/genitals/pustular

You can find more information about the spesolimab clinical studies here:

- Phase 1 study:
  https://clinicaltrials.gov/ct2/show/NCT04399837
- Phase 2 study (Effisayil™ 1):
  https://www.clinicaltrials.gov/ct2/show/NCT03782792

The full title of this article is:
Spesolimab for Generalized Pustular Psoriasis: A Review of Key Clinical Trials Supporting Initial US Regulatory Approval

You can find the full article here:
https://doi.org/10.3389/fimmu.2024.1359481
You can access the full article for free.

This summary was prepared by Kayleigh Walker and Debra Brocksmith of Envision Pharma Group. Plain language summary services were funded by Boehringer Ingelheim Pharmaceuticals, Inc. The summary was reviewed by Eran Gwillim and Anna Nichols, who are the authors of the original article.
